# Influence of BMI, Cigarette Smoking and Cryopreservation on Tyrosine Phosphorylation during Sperm Capacitation

**DOI:** 10.3390/ijms25147582

**Published:** 2024-07-10

**Authors:** Ana Ortiz-Vallecillo, Esther Santamaría-López, Diego García-Ruiz, David Martín-Lozano, Luz Candenas, Francisco M. Pinto, Manuel Fernández-Sánchez, Cristina González-Ravina

**Affiliations:** 1 IVIRMA Global Research Alliance, IVI Foundation, Instituto de Investigación Sanitaria La Fe, Avenida Fernando Abril Martorell, 106-Torre A, Planta 1ª, 46026 Valencia, Spain; ana.ortiz@ivirma.com (A.O.-V.); cristina.gonzalez@ivirma.com (C.G.-R.); 2 VIDA RECOLETAS Seville, Calle Américo Vespucio, 19, 41092 Seville, Spain; esther.santamaria@gruporecoletas.com; 3 Instituto de Investigaciones Químicas, CSIC, Calle Américo Vespucio, 49, 41092 Seville, Spain; david.martin@iiq.csic.es (D.M.-L.); luzcandenas@iiq.csic.es (L.C.); francisco.pinto@iiq.csic.es (F.M.P.); 4 Departamento de Cirugía, Universidad de Sevilla, Avenida Sánchez Pizjuán, S/N, 41009 Seville, Spain; 5 Departamento de Biología Molecular e Ingeniería Bioquímica, Universidad Pablo de Olavide, Carretera de Utrera, 1, 41013 Seville, Spain; 6 IVI-RMA Global Headquarters, Calle Américo Vespucio, 5, 41092 Seville, Spain

**Keywords:** BMI, overweight, cigarette, smoking, cryopreservation, freezing, capacitation, tyrosine phosphorylation, cryo-capacitation

## Abstract

Capacitation involves tyrosine phosphorylation (TP) as a key marker. Lifestyle-related factors, such as obesity and smoking, are recognized for their adverse effects on semen quality and male fertility, yet the underlying mechanisms, including their potential impact on TP, remain unclear. Moreover, the effect of sperm cryopreservation on TP at the human sperm population level is unexplored. Flow cytometry analysis of global TP was performed on pre-capacitated, post-capacitated and 1- and 3-hours’ incubated fresh and frozen–thawed samples from sperm donors (*n* = 40). Neither being overweight nor smoking (or both) significantly affected the percentage of sperm showing TP. However, elevated BMI and smoking intensity correlated with heightened basal TP levels (r = 0.226, *p* = 0.003) and heightened increase in TP after 3 h of incubation (r = 0.185, *p* = 0.017), respectively. Cryopreservation resulted in increased global TP levels after capacitation but not immediately after thawing. Nonetheless, most donors’ thawed samples showed increased TP levels before and after capacitation as well as after incubation. Additionally, phosphorylation patterns in fresh and frozen–thawed samples were similar, indicating consistent sample response to capacitation stimuli despite differences in TP levels. Overall, this study sheds light on the potential impacts of lifestyle factors and cryopreservation on the dynamics of global TP levels during capacitation.

## 1. Introduction

The process of capacitation is essential for male fertility. In the early 1950s, Austin and Chang first described the capacitation phenomenon and discovered that spermatozoa needed to spend a certain period of time within the female reproductive system to acquire fertilization potential [1,2,3,4]. In vitro sperm capacitation can be easily achieved with adequate culture conditions and medium composition to support the molecular and biochemical changes that occur during this process [5,6,7]. Currently, in vitro sperm capacitation serves not only as a diagnostic test but also as a preparatory step for intrauterine insemination (IUI) or in vitro fertilization (IVF). The two most commonly used methods for sperm preparation are swim-up and density gradient centrifugation (DGC) [8], with both techniques demonstrating comparable effectiveness in terms of reproductive outcomes [9].

Sperm capacitation is a complex process that remains largely unexplored. This involves precisely regulated events that encompass intricate molecular and cellular mechanisms [10,11]. Several aspects of capacitation include modifications in the lipid composition of the membrane; alterations in surface protein expression; elevations of intracellular Ca^2+^, cAMP, and pH; hyperpolarization of the membrane potential; and phosphorylation of threonine, serine and tyrosine residues [12,13]. All these events are important to achieve sperm hyperactivation and the acrosomal reaction, the purpose of which is membrane fusion with the oocyte and subsequent fertilization.

Notably, tyrosine phosphorylation (TP) is regulated through various molecular signal transduction pathways, with the cAMP-protein kinase A (PKA) pathway playing a pivotal role [13,14,15]. Activators and inhibitors of the cAMP-PKA pathway directly influence TP levels in spermatozoa [16]. TP has been identified as a crucial intracellular signaling event in the capacitation and regulation of sperm function [17]. Therefore, the assessment of TP levels has traditionally been considered a meaningful indicator for evaluating the capacitation status of spermatozoa [18].

Understanding the influence of lifestyle-related factors on sperm quality and fertility is essential to improve male reproductive health. Previous research has highlighted that obesity and smoking can negatively affect semen quality and male fertility and that both may be considered risk factors for male infertility [19,20], yet the underlying mechanisms remain unclear [21]. Additionally, it is important to further investigate the effects of common laboratory procedures, such as cryopreservation, on seminal samples at the molecular level, as these processes can induce significant stress and damage to spermatozoa [22].

Obesity in male partners can result in infertility and a lower likelihood of achieving a live birth through assisted reproductive technology, with several mechanisms proposed to explain the negative impact on male fertility [23,24]. These mechanisms include disorders of the hypothalamic–pituitary–gonadal axis, alterations in spermatogenesis, increased oxidative stress and adipokines produced by adipose tissue, and thermal effects resulting from increased scrotal adiposity [25]. Obesity can lead to declines in sperm concentration, motility, and morphology as well as changes in the acrosome reaction, membrane lipids, epigenetic print of spermatozoa, and DNA damage [26,27,28]. However, the impact of obesity on sperm quality and reproductive outcomes may vary and be influenced by other comorbid conditions [29,30].

There is controversy in the literature regarding the effects of smoking on semen parameters in fertile and infertile men, despite evidence linking cigarette smoking to decreased male fertility [31,32]. Molecular changes, including genetic and epigenetic modifications, have been proposed to mediate the association between smoking and impaired sperm functionality [31,33].

Spermatozoa cryopreservation is a widely used technique in ART programs, but it is associated with significant cryodamage, which can affect the quality of thawed samples [22]. Cryodamage primarily arises from temperature fluctuations, ice crystal formation, and osmotic stress resulting from the addition and removal of cryoprotective agents [34,35]. Freezing and thawing of sperm induce capacitation-like changes, often referred to as ‘cryo-capacitation’, which may affect sperm fertility [36].

While the effects of nicotine on murine spermatozoa have been linked to changes in TP levels [37], obesity in humans has been associated with increased levels and activity of a protein-tyrosine phosphatase [38]. Based on this evidence, we hypothesized that lifestyle-related factors might affect TP levels during sperm capacitation. Much information is available on the impact of cryopreservation, which has been shown to increase TP levels in various mammalian species [36], including humans [39]. Despite these insights, a comprehensive understanding of TP changes at the population level in human samples after cryopreservation is lacking. Therefore, this study aimed to investigate the influence of BMI, smoking and cryopreservation on the global TP levels in a sperm donor population.

## 2. Results

Donors were categorized according to their BMI as follows: underweight (BMI < 18.5 kg/m^2^) (n = 0), normal weight (BMI 18.5 to 24.9) (n = 26), overweight (BMI 25 to 29.9) (n = 14) and obese (BMI ≥ 30) (n = 0). With regard to smoking habits, donors were classified into two groups: smokers (n = 12) and non-smokers (n = 28). A total of 32 samples were used to investigate the impact of cryopreservation on global TP levels.

### 2.1. Influence of BMI on TP

Two BMI groups were compared: normal weight (n = 26) and overweight (n = 14), as none of the donors in the study was classified as underweight or obese. Age, cigarettes smoked per day, and fresh and capacitated seminal sample characteristics were similar between normal weight and overweight males (Table 1). Donor age exhibited no significant correlation with phosphorylation variables, which included TP as well as absolute and relative changes in TP between different time-point measurements (*p* > 0.05 for all).

BMI showed a weak positive correlation with TP B-cap (Pearson’s r = 0.226, *p* = 0.003). However, no significant correlations were observed between BMI and TP levels after capacitation (A-cap: r = 0.126, *p* = 0.104; 1 h: r = 0.031, *p* = 0.695; and 3 h: r = −0.031, *p* = 0.691). Furthermore, BMI was not correlated with absolute or relative changes in TP during capacitation (*p* > 0.05 for all). The overweight condition did not lead to a significant variation in pre-capacitation, post-capacitation or incubation (1 h and 3 h) TP values. Additionally, it did not have a significant effect on absolute and fold changes of TP over time (Table 2).

### 2.2. Influence of Cigarette Smoking Habit on TP

When categorizing the samples based on the donors’ smoking habits, we observed comparable characteristics in fresh samples. Age and BMI were also similar between groups. However, non-smokers had a higher percentage of immotile spermatozoa (Table 1).

On examining correlations between the daily number of cigarettes smoked and the measured TP values, no significant associations were observed (B-cap: r = −0.129, *p* = 0.096; A-cap: r = −0.109, *p* = 0.162; 1 h: r = −0.112, *p* = 0.149; and 3 h: r = −0.058, *p* = 0.456). Likewise, correlations between the number of cigarettes and absolute changes in the percentage of phosphorylation across different time points were not statistically significant. Notably, a weak positive correlation was found between the number of cigarettes smoked and the fold change in TP after 3 h of incubation (r = 0.185, *p* = 0.017).

However, being a smoker did not significantly influence TP levels (B-cap, A-cap, 1 h or 3 h) nor absolute or fold changes in TP over time (Table 3).

### 2.3. Influence of BMI and Cigarette Smoking Habit on TP

Overweight and smoking effects on TP levels were not statistically significant when combined in the models. Additionally, these factors did not influence the absolute or fold changes in TP over time (Table 4).

### 2.4. Influence of Cryopreservation on TP

The phosphorylation levels of 32 donated samples were analyzed before and after freezing–thawing to investigate the influence of cryopreservation on TP. Motility patterns (percentage of progressive and immotile spermatozoa) were different before and after freezing–thawing. Regarding global TP levels, we observed significantly increased means in the cryopreserved sample after capacitation (A-cap, 1 h and 3 h), but not before capacitation (Table 5). Nonetheless, the majority of donors exhibited higher TP values following the freezing and thawing processes (B-cap, A-cap, 1 h and 3 h) than in their fresh samples (Table 6). The rise in TP values following capacitation and during incubation showed significant differences within both the fresh and thawed samples (*p* < 0.001 for all TP comparisons).

Our observations revealed a trend indicating that beyond certain basal levels of phosphorylation in the non-capacitated fresh samples, the values after thawing tended not to reach the levels attained before freezing. To identify the inflection point where this shift in trend occurred, we plotted B-cap TP values before and after cryopreservation, ordered by the magnitude of the observed increase from the greatest to the smallest. Subsequently, two tendency lines were constructed (non-cryopreserved samples: y = 0.151x + 0.645, R^2^ = 0.314; cryopreserved samples: y = −0.127x + 5.997, R^2^ = 0.129), and their cut-off point was calculated [(x, y) = (19.28, 3.55)] to identify the *y*-axis value (% TP) at which this change in trend materialized.

**Figure 1:**
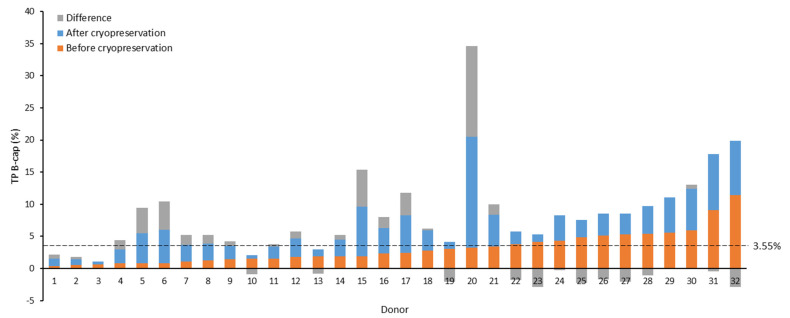
Effect of cryopreservation on tyrosine phosphorylation (TP) levels of non-capacitated samples from sperm donors. Seminal samples are arranged in ascending order based on TP values in the non-cryopreserved aliquot. The value of 3.55% (dotted line) represents the TP value obtained from the intersection of trend lines for cryopreserved and non-cryopreserved samples, ordered by the magnitude of the observed increase after freezing–thawing. B-cap: before capacitation; TP: tyrosine phosphorylation.

At the identified cut-off point (3.55%), we found that 81.0% (17/21) of non-capacitated samples exhibiting fresh TP values equal to or below the cut-off point displayed increased TP levels post-cryopreservation (Figure 1). In contrast, only 9.1% (1/11) of samples with elevated basal phosphorylation (>3.55%) showed increased levels after cryopreservation (B-cap: 81.0% (17/21) vs. 9.1% (1/11) *p* < 0.001). Similarly, a greater percentage of capacitated samples (A-cap) exhibited heightened levels after cryopreservation when their fresh basal phosphorylation levels were at or below 3.55% (A-cap: 90.5% (19/21) vs. 36.4% (4/11), *p* = 0.001). However, using this cut-off point revealed no significant differences in the proportion of samples that experienced increased TP levels after cryopreservation across the incubation periods (1 h: 46.9% (15/21) vs. 21.9% (7/11), *p* = 0.652; and 3 h: 66.7% (14/21) vs. 54.5% (6/11), *p* = 0.501).

**Figure 2:**
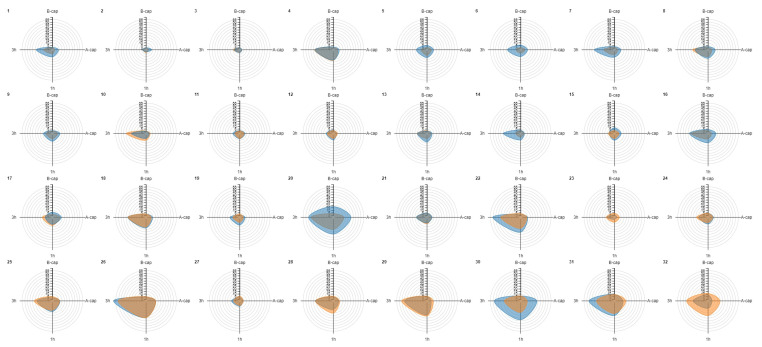
Radar chart representation for tyrosine phosphorylation (TP) values before and after cryopreservation for each donor sample. Orange denotes the fresh sample, while blue represents the frozen–thawed sample. The donors are ordered according to increasing levels of basal TP (B-cap) in the fresh sample. Notably, donors with lower baseline TP values in the fresh sample (B-cap) tended to achieve similar or higher levels TP after thawing, as evidenced by the predominance of the blue color at the top of the figure (e.g., donors 1 to 9). Conversely, those with higher fresh TP B-cap values generally did not exhibit a greater increase in TP values after cryopreservation, as indicated by the predominance of orange in the bottom of the graph (e.g., donors 28, 29 and 32). Similar patterns can be observed across most samples in the fluctuation of TP levels over processing time before and after cryopreservation (e.g., donors 4, 10 and 12, where very similar shapes for the fresh and cryopreserved conditions are evident). A-cap: after capacitation; B-cap: before capacitation; TP: tyrosine phosphorylation; 1 h: 1 h of incubation of the capacitated sample; 3 h: 3 h of incubation of the capacitated sample.

Radar charts were created for each donor sample to evaluate similarities in the increase in TP levels before and after cryopreservation. Interestingly, similar patterns were observed before and after cryopreservation in most samples (e.g., donors 4, 10 and 12) (Figure 2). In addition, it is worth noting that individuals with higher baseline TP (B-cap) in fresh samples did not experience the same extent of increase in their post-thaw levels as those with lower baseline levels, as mentioned above.

Following semen freezing–thawing, no significant differences were observed in TP (B-cap, A-cap, 1 h and 3 h) based on the donor’s body weight status, whether normal weight (n = 21) or overweight (n = 11) (3.1% vs. 2.9%, *p* = 0.531; 8.4% vs. 7.4%, *p* = 0.755; 10.7% vs. 10.9%, *p* = 0.434; and 21.3% vs. 25.4%, *p* = 0.876). Similarly, no differences were noted based on the donors’ smoking habits, whether non-smokers (n = 24) or smokers (n = 8) (3.3% vs. 2.8%, *p* = 0.728; 8.1% vs. 8.1%, *p* = 0.896; 11.5% vs. 10.9%, *p* = 0.811; and 21.8% vs. 29.4%, *p* = 0.654).

The mean cryostorage time was 181.3 days (min = 18 days, max = 486 days, standard deviation (SD) = 126.3 days). The time the sample was cryopreserved did not correlate significantly with TP values (B-cap, A-cap, 1 h and 3 h) (Table 7).

## 3. Discussion

The influence of tobacco use and obesity on seminal parameters and male fertility has been extensively researched [21]; however, there is no consensus on the mechanism(s) by which these conditions may affect male fertility. Given that TP is a critical signaling molecular modification during the capacitation process [40], we hypothesized that it might be affected in cases of elevated BMI and smokers. According to our study results, neither being overweight nor smoking appeared to have a significant impact on the percentage of sperm showing TP in sperm donors. Similarly, neither being overweight nor smoking had a significant effect on the post-thaw TP values.

However, our results showed that elevated BMI and smoking intensity could affect TP levels. We observed a positive correlation between BMI and basal phosphorylation values, indicating that individuals with an elevated BMI may have a higher number of spermatozoa showing TP in unprocessed fresh ejaculates. It is known that spermatozoa from an ejaculate undergo capacitation and acrosome reaction in different pools that extend the fertile window of the ejaculate [41]. Therefore, a premature increase in TP levels associated with capacitation may indicate a lower number of spermatozoa with fertilization potential at the time of fertilization. Regarding smoking, a positive correlation was detected between the number of cigarettes smoked per day and higher phosphorylation increments from the end of sample processing by DGC to the third hour of incubation. This suggests acceleration of capacitation in heavy smokers.

Limited information is currently available on the effects of being overweight/obesity and cigarette smoking on TP levels. It is known that the TP level at a given time during capacitation is influenced by the interplay between tyrosine kinases and phosphatases [42]. Shi et al. reported elevated levels and activity of a protein-tyrosine phosphatase in capacitated sperm obtained from obese patients compared to samples from non-obese donors. This was associated with prolonged dephosphorylation of a key regulator protein of vesicle fusion and was correlated with defects in the acrosome reaction [38]. These findings indicate that obesity may be associated with alterations in tyrosine-phosphorylated proteins, which could affect sperm fertility. Regarding smoking and TP, nicotine exposure, one of the most detrimental components of tobacco, resulted in a significant reduction in tyrosine-phosphorylated proteins in caudal epididymal spermatozoa of mice following in vitro capacitation compared to the non-treated group [37]. Therefore, our study represents the first attempt to elucidate the effects of these lifestyle-related factors on global levels of this marker in human semen samples.

Previous research has documented TP increases in multiple mammalian species after cryopreservation [36], including a rise in the TP of four sperm proteins from human donors [39]. However, this phenomenon should not be considered per se as a physiological event that prepares sperm for fertilization; rather, it would result in a reduction in the fertilization efficiency of the sperm population as a whole [43]. We observed a significant increase in the proportion of spermatozoa exhibiting TP at the population level following cryopreservation and capacitation, but not immediately after thawing. However, most donor samples (18/32) showed an increase in TP levels immediately after freezing–thawing. Interestingly, we observed that non-capacitated samples with higher fresh TP levels (>3.55%) tended to have similar or diminished TP levels before and immediately after capacitation following freezing-thawing in comparison with their TP values before freezing. However, regardless of the basal level of TP when fresh, after prolonged incubation periods under capacitating conditions, thawed samples generally exhibited increased TP with respect to their fresh values when capacitated and incubated. Ultimately, this could be related to fertility potential, as all samples resulting in pregnancy following IUI (3 of 32) had global fresh TP levels below the threshold and heightened levels after cryopreservation. Nevertheless, it is worth noting that the pattern of TP during cryopreservation is distinct from that induced during in vitro capacitation [36].

The phosphorylation patterns in fresh and frozen–thawed samples from the same individual were similar, which has not been reported previously. This finding suggests that the samples react consistently to the capacitation stimulus; however, as stated above, the levels of response were not identical to those prior to freezing. Furthermore, the duration of cryostorage was not significantly associated with phosphorylation levels. This aligns with existing research suggesting that while extended storage of seminal samples may result in a decrease in quality, the duration of cryostorage does not appear to have a significant impact on the success rates of IVF and IUI treatments that utilize donor sperm [44]. Further research with larger sample sizes is necessary to determine whether pre- and post-cryopreservation levels and patterns of TP can serve as indicators of positive reproductive prognosis in insemination treatments.

Historically, western blotting has been the most frequently used method for determining TP levels. However, this only provides an average value for the entire semen sample, which may mask crucial information owing to the complex and heterogeneous nature of the ejaculate. Additionally, standardizing this method between laboratories is difficult and time-consuming. Fluorescence microscopy is an alternative that has been utilized to identify the subcellular localization of TP and the proportion of cells showing phosphorylation; however, it has the limitation of examining only a limited number of cells [45,46]. In our study, we used flow cytometry for the overall estimation of TP associated with sperm capacitation in a seminal sample, an approach used for the first time by Sidhu et al. [47]. Flow cytometry provides quantitative data on the percentage of cells exhibiting TP, enabling a more detailed examination of cellular heterogeneity within the sample. In fact, it is suggested that two major platforms could be useful for future regular evaluations of TP: microscopy with the aid of computer-assisted image acquisition and analysis; and conventional flow cytometry or flow cytometry combined with image capture and analysis [48,49]. To the best of our knowledge, this is the first study to examine the influence of BMI and smoking habits on the global effect of TP in human semen samples using flow cytometry, and it is the first to employ this technique to analyze the effect of cryopreservation on sperm donor samples.

Among the limitations of our study, it is worth highlighting that we only included sperm donors with proven fertility, so we cannot extrapolate the results to other populations, such as infertile patients undergoing assisted reproduction. Additionally, we were unable to analyze the influence of extreme BMI values and could not study the effect of obesity on our study marker, as this was a reason for exclusion from the donor program. Therefore, the results must be confirmed in subsequent studies by expanding the study population and increasing sample size.

Combining both the molecular and clinical perspectives, this study provides novel insights into the potential influence of elevated BMI, smoking and cryopreservation on seminal quality and male reproductive health. In summary, the findings of this study suggest that being overweight and cigarette smoking do not have a significant impact on TP levels before or after capacitation. However, it is important to consider the potential influence of extreme BMI and high smoking status on TP levels. Additionally, we showed that the cryopreservation process is associated with an increase in TP at the population level after capacitation. These findings are based on a cohort of semen donors; therefore, additional research is warranted to determine whether similar trends exist in infertile patients.

## 4. Materials and Methods

### 4.1. Study Population

The study population and analyses presented in this manuscript are part of a prospective experimental unicentric study that received approval from the Institutional Ethics Committee of the University Hospitals Virgen Macarena and Virgen del Rocío (Seville, Spain) and was registered on Clinical Trials (www.clinicaltrials.gov, accessed on 29 June 2021) with the identifier NCT04962100.

Male donors providing a fresh semen sample were recruited for this study. A period of sexual abstinence lasting 2 to 5 days was required. The following criteria lead to the exclusion from this study: cryptozoospermia or oligozoospermia with a sperm concentration below 1 million per milliliter and ejaculates collected after more than 5 days of sexual abstinence. All men signed an informed consent form to participate in the study. During the study period, 167 fresh semen samples from sperm donors (n = 40) with proven fertility were included. At least 1 sample per donor, and up to a maximum of 5, were analyzed. A flowchart of the donors and donated samples subjected to analysis is shown in Figure S1.

### 4.2. Semen Analysis and Sperm Capacitation

Semen samples were obtained by masturbation and were incubated at 37 °C for 10 min until complete liquefaction. Samples were analyzed according to the World Health Organization guidelines [50]. The variables analyzed in the fresh sample were volume (mL), concentration (mill/mL), motility (progressive, non-progressive and immotile spermatozoa) (%), morphology (%) and vitality (%). Sperm concentration and motility were measured using a Makler Chamber (Sefi-Medical Instruments, Haifa, Israel). Morphology was evaluated after Diff-Quik staining (Panreac AppliChem ITW Reagents, Darmstadt, Germany) and vitality was assessed after eosin-nigrosin dye exclusion test (Sigma-Aldrich, Merck, Darmstadt, Germany), as well as both being assessed by optical microscopy.

Then, 0.4 mL of the sperm sample was capacitated by density gradient centrifugation (DGC) using a 3-layer Percoll^®^ density gradient (95–70–45%) protocol. First, semen samples were washed 1:2 (Ferticult^TM^ Flushing Medium, FertiPro, Beernem, Belgium) and centrifuged at 300× *g* for 5 min. To obtain 45%, 70% and 95% dilutions, Sil-Select STOCK^TM^ (Sil-Select STOCK^TM^, FertiPro, Beernem, Belgium) was diluted in Ferticult^TM^ Flushing Medium. Gradient columns were prepared by gently layering 1 mL of each solution in conical tubes, starting with the 95% solution at the bottom and followed by the 70% and 45% fractions. The washed samples were layered on top of the columns and processed by centrifugation at 300× *g* for 10–12 min. The recovered 95% pellet was resuspended in 1–2 mL of wash medium, centrifuged at 300× *g* for 5 min to eliminate colloidal particles, and finally resuspended in sperm culture medium (0.5 mL).

### 4.3. Sperm Cryopreservation and Intrauterine Insemination

The remaining fresh semen sample that was not subjected to TP assessment was mixed with SpermFreeze^TM^ SSP (FertiPro, Beernem, Belgium) at 3:1 ratio. Sperm cryopreservation medium was added drop by drop while gently swirling. Subsequently, the mixture was transferred to cryotubes and allowed to equilibrate for 45 min at 4 °C. Following this, it underwent further equilibration with nitrogen vapor at various heights (5 and 10 cm) above the liquid nitrogen surface for an additional hour at each height. Finally, the cryotubes were immersed in liquid nitrogen (−196 °C) and stored in a vapor-phase liquid nitrogen tank.

All donated samples were cryopreserved for future utilization in assisted reproductive cycles. A seminal sample was meticulously chosen from each donor (adhering to the post-thaw seminal quality criteria established by the clinic) for IUI procedures involving donor sperm. The selected samples were then utilized in IUI cycles, enabling the analysis of TP levels following thawing. Of the 40 potential samples that could have been used, 32 were finally utilized in IUI cycles during the study period, whereas the remaining 8 samples did not meet the required post-thaw quality criteria.

Fresh samples included in the study that were assigned to IUI treatments were thawed by first placing the cryotubes in water at room temperature for 10 min and then in a thermoblock at 37 °C for 30 min. Once the thawing process was completed, the sample was capacitated according to the procedure described above. Aliquots were obtained during the process to measure TP. The IUI protocol followed has been described elsewhere [51].

### 4.4. Tyrosine Phosphorylation (TP) Assessment

TP analysis was performed using flow cytometry, following previously described procedures [52,53]. For each sample, the percentage of phosphorylated tyrosine residues was assessed at four distinct time points: prior to capacitation (B-cap), immediately following capacitation (A-cap) and after 1 h (1 h) and 3 h (3 h) of post-capacitation incubation at 37 °C under capacitating conditions. TP was measured both before freezing and after freezing–thawing in samples intended for IUI. Therefore, we collected phosphorylation data for identical samples both before and after the cryopreservation procedure, encompassing both pre-capacitation and post-capacitation stages. Absolute and relative changes (fold changes) were calculated between the different TP measurement points (B-cap to A-cap, A-cap to 1 h, A-cap to 3 h, and 1 h to 3 h).

At the times indicated, spermatozoa were fixed in 4% paraformaldehyde and permeabilized in 0.2% Triton X-100 for 10 min at room temperature. The samples were then incubated in PBS with a mouse monoclonal antibody designed to recognize human tyrosine phosphoproteins and conjugated with Alexa Fluor 488 (sc-508 AF488, Santa Cruz Biotechnology, Santa Cruz, CA, USA) at a 1:200 dilution for 1 h. In parallel, an aliquot of B-cap and A-cap were incubated only in PBS to serve as a control of autofluorescence.

Fluorescence data from at least 20,000 events per sample were captured on a BD Accuri C6 flow cytometer (BD Biosciences, San José, CA, USA). Green fluorescence was collected in the FL1 sensor and analyzed using CFlow Plus software. Fluorescence values obtained are reported as percentage of TP (%).

### 4.5. Statistical Analysis

The values of all samples from the same donor were averaged to obtain a single representative value for each donor for descriptive analysis. Donors were categorized based on their BMI (according to the World Health Organization (WHO) guidelines [54]) and smoking habits (smoker or non-smoker). To analyze the effect of cryopreservation on TP levels (samples designated for IUI), they were categorized as either non-cryopreserved or cryopreserved.

Differences between cohorts (classified according to BMI or smoking habits) were assessed using the Student’s *t*-test, while differences between paired samples (data before and after cryopreservation) were assessed using the paired *t*-test. The Mann–Whitney U test was used to compare cohorts after freezing–thawing. The chi-squared (X^2^) test was employed to compare proportions, except when the expected frequencies were <5, in which case Fisher’s exact test was utilized. We used generalized estimating equation (GEE) models to address the repeated-measures structure, accounting for within-subject correlation and examining the impact of BMI and/or smoking on our outcome variables. Pearson’s r or Spearman’s rho tests were used for correlation analyses, depending on the normality of the data. Data are presented as mean and 95% confidence intervals (CI). In all cases, a *p* value < 0.05 was considered significant. Statistical analysis was performed using R Statistical Software (v4.3.0, Vienna, Austria) [GEE models] and IBM SPSS Statistics Version 27.0 (IBM Corp., Armonk, NY, USA) [rest of statistical analysis].

## Figures and Tables

**Table 1:** Fresh and capacitated seminal sample characteristics according to BMI and cigarette smoking.

	Classification According to BMI (n = 40)			Classification According to Cigarette Smoking Habit (n = 40)			Overall (n = 40)
Variable	Normal Weight (n = 26)	Overweight (n = 14)	*p*-Value	Non-Smokers (n = 28)	Smokers (n = 12)	*p*-Value	
Age	24.8 (22.7–26.8)	26.1 (21.3–30.9)	0.539	24.4 (22.2–26.6)	27.2 (22.4–31.9)	0.220	25.3 (23.4–27.3)
BMI	21.8 (21.2–22.5)	27.0 (26.1–27.9)	<0.001*	23.7 (22.5–24.8)	23.7 (21.6–25.7)	1.000	23.7 (22.7–24.6)
Cigarettes smoked daily (cig/day)	1.5 (0.3–2.7)	2.1 (−0.3–4.6)	0.580	0.0 (0.0–0.0)	5.7 (3.2–8.4)	<0.001 *	1.73 (0.6–2.8)
***Fresh seminal sample***							
Sexual abstinence (day)	3.5 (3.3–3.6)	3.4 (3.2–3.7)	0.886	3.4 (3.3–3.6)	3.5 (3.2–3.7)	0.885	3.4 (3.3–3.6)
Volume (mL)	3.0 (2.6–3.5)	3.0 (2.5–3.6)	0.968	3.2 (2.8–3.6)	2.7 (2.0–3.4)	0.192	3.0 (2.7–3.4)
Progressive motility (%)	66.0 (63.3–68.6)	66.4 (62.7–70.1)	0.850	66.2 (63.4–69.0)	66.0 (63.4–68.7)	0.944	66.1 (64.1–68.2)
Non-progressive motility (%)	7.0 (6.2–7.7)	6.1 (5.0–7.1)	0.147	6.6 (5.9–7.3)	6.7 (5.5–8.0)	0.867	6.6 (6.0–7.2)
Immotile (%)	27.1 (24.6–29.5)	27.6 (24.0–31.1)	0.807	27.2 (24.7–29.7)	27.3 (24.1–30.5)	0.982	27.2 (25.3–29.2)
Vitality (%)	78.4 (75.9–81.0)	76.6 (72.6–80.5)	0.389	77.7 (75.1–80.3)	78.1 (74.1–82.1)	0.857	77.8 (75.7–79.9)
Normal forms (%)	9.8 (9.0–10.5)	9.9 (9.1–10.8)	0.812	10.0 (9.3–10.7)	9.4 (8.4–10.3)	0.259	9.8 (9.3–10.4)
TSC (mill)	219.5 (193.9–245.1)	201.1 (164.4–237.8)	0.388	222.2 (199.1–245.3)	191.6 (147.8–235.5)	0.164	213.0 (192.8–233.3)
TPMSC (mill)	146.6 (127.7–165.5)	135.5 (109.0–162.0)	0.477	148.5 (131.7–265.3)	129.3 (96.4–162.2)	0.233	142.7 (127.9–157.5)
***Capacitated seminal sample***							
Progressive motility (%)	90.0 (88.6–91.4)	90.6 (88.5–92.6)	0.617	90.8 (89.5–92.2)	88.7 (86.7–90.6)	0.071	90.2 (89.1–91.3)
Non-progressive motility (%)	3.0 (2.4–3.7)	2.6 (2.1–3.1)	0.293	2.8 (2.4–3.3)	3.0 (1.8–4.1)	0.795	2.9 (2.4–3.3)
Immotile (%)	7.0 (5.8–8.2)	6.1 (4.7–7.5)	0.368	6.0 (4.9–7.0)	8.4 (6.8–10.0)	0.013 *	6.7 (5.8–7.6)
Normal forms (%)	11.5 (10.6–12.3)	11.3 (10.6–11.9)	0.778	11.8 (11.0–12.4)	10.6 (9.6–11.6)	0.064	11.4 (10.8–12.0)
TSC (mill)	124.5 (103.6–145.3)	96.7 (64.9–128.4)	0.12	119.1 (97.1–141.2)	104.5 (74.9–134.1)	0.438	114.7 (97.5–131.9)
TPMSC (mill)	113.2 (93.4–133.1)	88.6 (59.1–118.2)	0.145	109.3 (88.5–130.2)	93.6 (66.8–120.5)	0.375	104.6 (88.4–120.8)

BMI: body mass index; TSC: total sperm count; TPMSC: total progressive motile sperm count. Results are indicated by mean and 95% confidence interval. The data from different semen samples from the same donor were averaged. Student’s *t*-test was employed to compare the groups (normal weight vs. overweight and smokers vs. non-smokers). * Statistically significant difference (*p* < 0.05).

**Table 2:** Overweight influence on tyrosine phosphorylation (TP).

Outcome Variable	Estimated Coefficient	Standard Error	*p*-Value
***TP* (%)**			
B-cap	0.68	0.35	0.27
A-cap	0.49	1.34	0.72
1 h	−0.32	2.14	0.88
3 h	−1.26	3.85	0.74
***Absolute changes in TP* (%)**			
B-cap to A-cap	−0.18	0.99	0.85
A-cap to 1 h	−0.81	1.12	0.47
A-cap to 3 h	−1.75	3.14	0.58
1 h to 3 h	−0.94	2.21	0.67
***Fold change in TP***			
B-cap to A-cap	−0.47	0.57	0.41
A-cap to 1 h	−1.33	0.87	0.13
A-cap to 3 h	−1.21	1.08	0.27
1 h to 3 h	−0.18	0.24	0.47

A-cap: after capacitation; B-cap: before capacitation; TP: tyrosine phosphorylation; 1 h: 1 h of incubation of the capacitated sample; 3 h: 3 h of incubation of the capacitated sample. Generalized estimating equation (GEE) models were used.

**Table 3:** Influence of cigarette smoking on tyrosine phosphorylation (TP).

Outcome Variable	Estimated Coefficient	Standard Error	*p*-Value
***TP* (%)**			
B-cap	−0.07	0.63	0.92
A-cap	−0.4	1.4	0.78
1 h	−2.47	2.19	0.27
3 h	−5.67	3.9	0.15
***Absolute changes in TP* (%)**			
B-cap to A-cap	−0.33	1.02	0.75
A-cap to 1 h	−2.07	1.13	0.07
A-cap to 3 h	−5.27	3.17	0.10
1 h to 3 h	−3.20	2.25	0.16
***Fold change in TP***			
B-cap to A-cap	0.37	0.59	0.54
A-cap to 1 h	0.37	0.93	0.70
A-cap to 3 h	0.45	1.12	0.69
1 h to 3 h	−0.02	0.25	0.92

A-cap: after capacitation; B-cap: before capacitation; TP: tyrosine phosphorylation; 1 h: 1 h of incubation of the capacitated sample; 3 h: 3 h of incubation of the capacitated sample. Generalized estimating equation (GEE) models were used.

**Table 4:** Overweight and cigarette smoking influence on tyrosine phosphorylation (TP).

	Overweight			Cigarette Smoker		
Outcome Variable	Estimated Coefficient	Standard Error	*p*-Value	Estimated Coefficient	Standard Error	*p*-Value
***TP* (%)**						
B-cap	0.11	0.10	0.27	−0.07	0.63	0.92
A-cap	0.02	0.22	0.94	−0.4	1.42	0.78
1 h	−0.04	0.35	0.92	−2.47	2.22	0.27
3 h	−0.18	0.62	0.77	−5.67	3.95	0.16
***Absolute changes in TP* (%)**						
B-cap to A-cap	−0.09	0.16	0.57	−0.33	1.03	0.75
A-cap to 1 h	−0.05	0.18	0.77	−2.07	1.14	0.08
A-cap to 3 h	−0.2	0.51	0.69	−5.27	3.21	0.11
1 h to 3 h	−0.15	0.36	0.68	−3.2	2.27	0.17
***Fold change in TP***						
B-cap to A-cap	−0.12	0.09	0.19	0.37	0.59	0.54
A-cap to 1 h	−0.15	0.15	0.32	0.36	0.93	0.70
A-cap to 3 h	0.01	0.18	0.95	0.45	1.13	0.69
1 h to 3 h	−0.01	0.04	0.85	−0.02	0.25	0.92

A-cap: after capacitation; B-cap: before capacitation; TP: tyrosine phosphorylation; 1 h: 1 h of incubation of the capacitated sample; 3 h: 3 h of incubation of the capacitated sample. Generalized estimating equation (GEE) models were used.

**Table 5:** Effect of seminal cryopreservation on motility patterns and tyrosine phosphorylation (TP) levels.

	Non-Cryopreserved Sample (n = 32)	Cryopreserved Sample (n = 32)	*p*-Value
***Non-capacitated sample***			
Progressive motility (%)	68.6 (65.7–71.5)	36.0 (32.2–39.8)	<0.001 *
Non-progressive motility (%)	6.2 (4.8–7.6)	6.6 (4.6–8.5)	0.758
Immotile (%)	25.2 (22.7–27.8)	57.3 (52.4–62.1)	<0.001 *
Normal forms (%)	10.6 (9.4–11.8)	9.7 (8.8–10.7)	0.274
***Capacitated sample***			
Progressive motility (%)	91.1 (89.3–92.9)	81.7 (78.7–84.7)	<0.001 *
Non-progressive motility (%)	3.3 (2.2–4.4)	4.4 (2.9–5.9)	0.284
Immotile (%)	5.7 (4.4–6.9)	13.8 (11.1–16.5)	<0.001 *
Normal forms (%)	11.8 (10.7–12.9)	11.6 (10.6–12.5)	0.731
***TP* (%)**			
B-cap	3.1 (2.2–4.0)	3.9 (2.7–5.1)	0.180
A-cap	6.7 (4.9–8.4)	8.9 (6.7–11.2)	0.035 *
1 h	11.2 (8.5–13.9)	13.3 (10.4–16.2)	0.032 *
3 h	21.1 (16.3–25.9)	24.8 (19.7–30.0)	0.026 *
***Absolute changes in TP* (%)**			
B-cap to A-cap	3.5 (2.3–4.7)	5.0 (3.3–6.8)	0.090
A-cap to 1 h	4.5 (2.9–6.2)	4.4 (2.4–6.4)	0.820
A-cap to 3 h	14.5 (10.5–18.4)	15.9 (11.4–20.4)	0.339
1 h to 3 h	9.9 (7.3–12.6)	11.5 (8.5–14.5)	0.194
***Fold change in TP***			
B-cap to A-cap	2.6 (2.1–3.2)	3.1 (2.3–3.9)	0.280
A-cap to 1 h	1.9 (1.6–2.2)	1.7 (1.4–2.1)	0.409
A-cap to 3 h	4.2 (3.0–5.4)	3.7 (2.8–4.7)	0.474
1 h to 3 h	2.0 (1.7–2.3)	2.3 (1.7–3.0)	0.340

A-cap: after capacitation; B-cap: before capacitation; TP: tyrosine phosphorylation; 1 h: 1 h of incubation of the capacitated sample; 3 h: 3 h of incubation of the capacitated sample. Results are indicated by mean and 95% confidence interval. Paired *t*-test was employed to compare variables before and after freezing–thawing. * Statistically significant difference (*p* < 0.05).

**Table 6:** Donors’ tendency to decrease, increase or maintain tyrosine phosphorylation (TP) levels after cryopreservation compared to fresh samples.

	B-Cap	A-Cap	1 h	3 h	Total
**Increase**	18 (56.3)	22 (68.8)	22 (68.8)	20 (62.5)	82 (64.1)
**Decrease**	14 (43.7)	9 (28.1)	10 (31.2)	12 (37.5)	45 (35.2)
**No change**	0 (0.0)	1 (3.1)	0 (0.0)	0 (0)	1 (0.7)
**Total**	32 (100)	32 (100)	32 (100)	32 (100)	128 (100)

A-cap: after capacitation; B-cap: before capacitation; TP: tyrosine phosphorylation; 1 h: 1 h of incubation of the capacitated sample; 3 h: 3 h of incubation of the capacitated sample. Data presented as n (%).

**Table 7:** Correlations between cryostorage time and tyrosine phosphorylation (TP).

Variable 1	Variable 2	Spearman’s Rho	*p*-Value
Cryostorage time (days)	TP B-cap (%) after F–T	−0.138	0.453
	TP A-cap (%) after F–T	−0.148	0.420
	TP 1 h (%) after F–T	−0.175	0.337
	TP 3 h (%) after F–T	0.030	0.869

A-cap: after capacitation; B-cap: before capacitation; F–T: freezing–thawing; TP: tyrosine phosphorylation; 1 h: 1 h of incubation of the capacitated sample; 3 h: 3 h of incubation of the capacitated sample. Spearman correlation coefficient (rho) was used for correlation calculations.

## Data Availability

The raw data supporting the conclusions of this article will be made available by the authors on request.

## Supplementary Materials

The following supporting information can be downloaded at: https://www.mdpi.com/article/10.3390/ijms25147582/s1.
