# Supplementary material for: Influence of BMI, Cigarette Smoking and Cryopreservation on Tyrosine Phosphorylation during Sperm Capacitation
Source: Int J Mol Sci. 2024 Jul 10;25(14):7582. doi: 10.3390/ijms25147582 (PMC11276716; doi:10.3390/ijms25147582)
Supplement: Supplementary file 1 [file ijms-25-07582-s001.zip › ijms-2986165-supplementary.pdf]

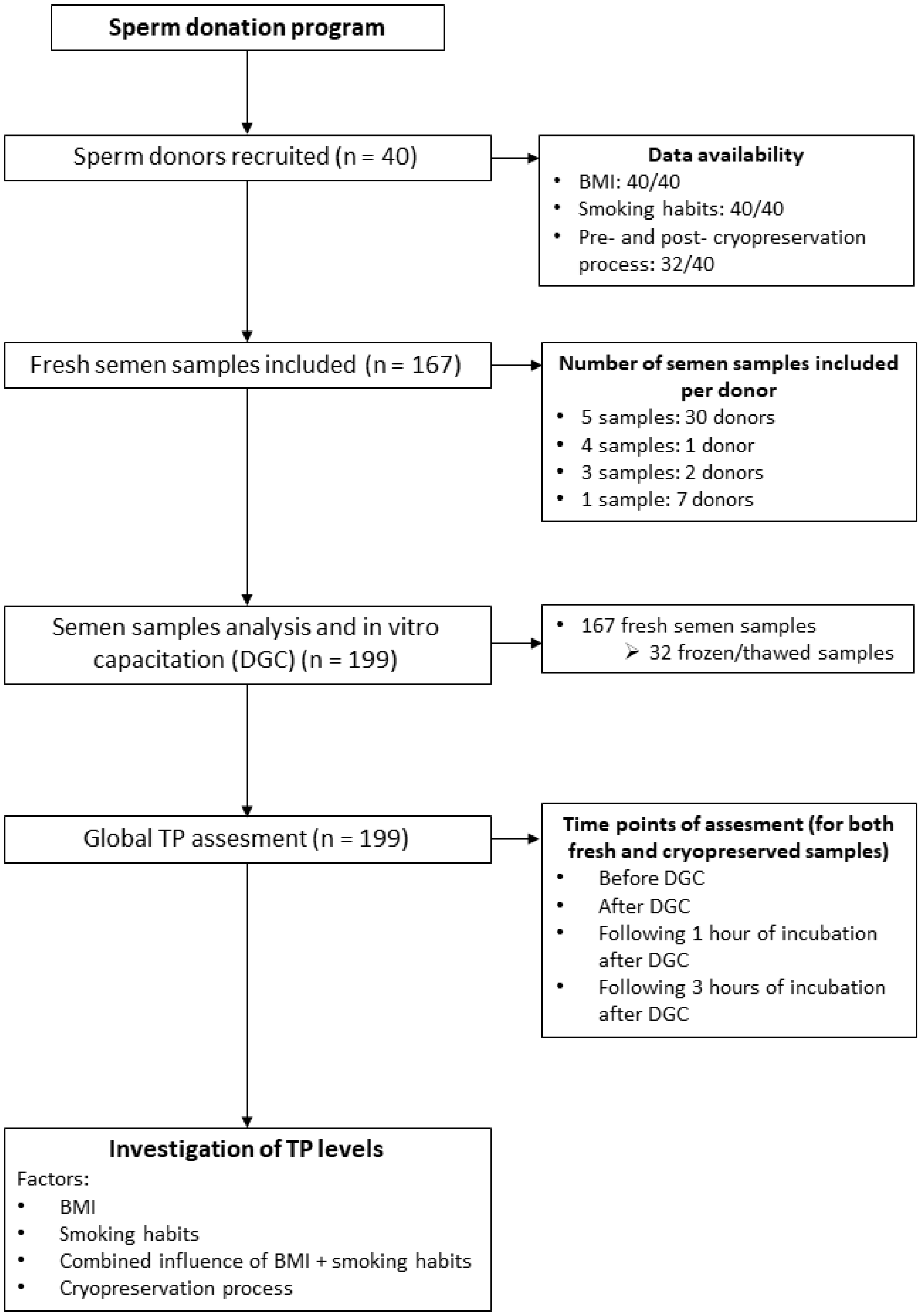

Figure S1. Flowchart of donors and donated samples included in the study. BMI: body mass index, DGC: density gradient centrifugation, TP: tyrosine phosphorylation.
